# Cariogenic Dietary Assessment Using a Mobile App in Children

**DOI:** 10.3290/j.ohpd.c_1846

**Published:** 2025-02-20

**Authors:** Matina V. Angelopoulou, Andreas Agouropoulos, Niklas Palaghias, Philippos Orfanos, Vasiliki Benetou, Christos Rahiotis, Sotiria Gizani

**Affiliations:** a Matina V. Angelopoulou Pediatric Dentist, Clinical Instructor, Department of Pediatric Dentistry, School of Dentistry, National and Kapodistrian University of Athens, Athens, Greece. Designed the app, conducted the study, drafted the manuscript, read and approved the final version of the manuscript.; b Andreas Agouropoulos Assistant Professor, Department of Pediatric Dentistry, School of Dentistry, National and Kapodistrian University of Athens, Athens, Greece. Contributed to the design of the app and edited the manuscript, read and approved the final version of the manuscript.; c Niklas Palaghias Electrical Engineer, CEO Quadible, London. Developed the app, read and approved the final version of the manuscript.; d Philippos Orfanos Biostatistician-Epidemiologist, Department of Hygiene, Epidemiology and Medical Statistics, School of Medicine, National and Kapodistrian University of Athens, Athens, Greece. Performed the statistical analysis, read and approved the final version of the manuscript.; e Vasiliki Benetou Professor, Department of Hygiene, Epidemiology and Medical Statistics, School of Medicine, National and Kapodistrian University of Athens, Athens, Greece. Contributed to the dietary assessment and study design, edited the manuscript, read and approved the final version of the manuscript.; f Christos Rahiotis Associate Professor, Department of Operative Dentistry, School of Dentistry, National and Kapodistrian University of Athens, Athens, Greece. Interpreted the results, edited the manuscript, read and approved the final version of the manuscript.; g Sotiria Gizani Associate Professor and Chair, Department of Pediatric Dentistry, School of Dentistry, National and Kapodistrian University of Athens, Athens, Greece. Inspired the study’s concept/idea, contributed to all steps of the study, critically revised the manuscript, read and approved the final version of the manuscript.

**Keywords:** dental office, dietary advice, dietary assessment, mobile application

## Abstract

**Purpose:**

A direct association exists between caries and high-sugar diets. The aim of this study was to test whether the cariogenicity of diet assessed and analysed through a new mobile app is associated with caries risk among children.

**Materials and Methods:**

A total of 247 children, 2–15 years old, were recruited from university and hospital daycare centers and dental clinics. Diet was assessed via a 24-h recall, and caries (dmft/DMFT- ICDAS criteria) was documented through clinical examination. A mobile app was designed to analyse dietary data and calculate the diet’s cariogenicity. Demographics, daily meals, meal duration and type, and toothbrushing were entered. A diagram presenting an estimate of the oral pH was produced, showing the time interval during which caries may develop, and a calculated cariogenic dietary risk was generated. Multivariate logistic regression derived odds ratios estimating associations between the cariogenic diet and caries across three age groups.

**Results:**

A cariogenic diet analysed by the app was positively associated with dfmt (r = 0.477, p < 0.001) in 2- to 6-year-old children, with dmft (r = 0.376, p < 0.05) and DMFT (r = 0.271, p < 0.05) in 7- to 11-year-old children, and with DMFT (r = 0.383, p < 0.001) in 12- to 15-year-old children. Parents’ lower educational level was associated with a statistically significantly higher caries risk in younger children (p < 0.05).

**Conclusion:**

Cariogenic diet evaluated with the new app was associated with increased caries, providing evidence of an accurate assessment among children. This app could assist dentists in providing dietary assessment and advice related to caries risk at the dental office in a more structured, educational, and time-saving way.

Unhealthy diet has been considered a common risk factor for numerous diseases, and regarding oral health, it has been directly associated with caries.^4^ More specifically, frequent sugar consumption has been found to increase caries incidence, and in developed countries, increased consumption of refined carbohydrates increases caries in the population.^32^

Despite the proven association of caries with diet, the number of tools for analysing diet in a dental setting is limited, as is time during the visit. As a result, dentists rarely perform such analyses in daily practice.^25^

Nevertheless, numerous dietary assessment tools are available for the measurement and evaluation of habitual diet, both for professionals and for individual use.^9^ These tools include conventional dietary assessment methods, computer software, and mobile applications (apps). However, there are few tools to evaluate dietary data for cariogenicity. The analysis of the daily minute score of acid production was the first countable method used in dental clinical practice.^28^ Some studies have suggested tools for computing a score based on the frequency of consumption of low and high cariogenic food and drinks, while others analyse a cariogenic diet based on the daily weight or frequency of sugar consumption, or alternatively, the daily calorie-intake from sugars.^5,10,28^ Moreover, the Healthy Eating Index (HEI), a dietary index measuring adherence to specific dietary guidelines, has also been used to detect associations between diet and caries risk in childhood.^29,37^

Mobile technology has invaded everyday life, and multiple apps have been developed to promote public health.^15^ In dentistry, most apps focus on oral hygiene promotion.^31^ Additionally, overall caries-risk tools are available in app form,^8,30^ while dental trauma apps,^2,19^ and apps for orthodontic patients can also be found.^21,33,35^ There is one app that provides information on cariogenic diet,^1^ and one that provides a Food Diary platform to report daily diet (FoodForTeeth). Recently, an app for dental dietary assessment was published, which underlines the need for developing such tools globally.^16^ However, these apps have not been validated and none have been tested in children.

Informing and educating dental patients on the ways diet and eating habits contribute to caries formation is essential to protect and improve their oral health. If cariogenic dietary habits are not modified, especially in high-risk patients, caries will continue to rise over their lifetime. Therefore, if a user-friendly tool is not available to detect harmful dietary habits in the dental setting, then the ability to obtain individualised dietary advice would be limited. Thus, the use of an app to calculate diet’s cariogenicity, especially in younger children, may be useful. Indeed, by the use of such an app, individuals can identify the unhealthy dietary behaviours that need to be changed to improve their oral health. The proposed application was developed with the aim of being used as a diagnostic tool for diet’s cariogenicity in the daily dental practice for the design of an individualised preventive program. Also, it can be used as an education and communication tool for the patient, who can visualise the impact of his/her efforts to change their diet in terms of decreasing their caries risk.

The purpose of this study was to develop a new mobile app for cariogenic diet assessment and analysis based on the available scientific evidence and to test its accuracy in a sample of children from different age groups.

## Materials and Methods 

### Study Design

This was a cross-sectional study using a mobile app to assess dietary habits and their relation to caries in children. Children’s dietary habits and demographic characteristics were evaluated via a questionnaire. Caries was assessed through clinical examination. This study was approved by the Ethics Committee of the School of Dentistry, National and Kapodistrian University of Athens (No 514/21.06.2022).

Initially, scientific evidence on dietary assessment and analysis was collected to help design the application. The main principle of this app was based on the fact that an oral pH-drop below 5.5 (critical pH), which occurs after each meal, encourages enamel demineralisation and thus caries formation.^22^ Oral pH is strongly influenced by sugar intake, which is represented by the graph produced by the application. The application was designed by a computer engineer and was tested for its applicability with the parents of the young patients of the Paediatric Dentistry Postgraduate Clinic, National and Kapodistrian University of Athens.

### Study Sample

A total of 247 children, 2–15 years old, were recruited from the paediatric and orthodontic dental clinics of the National and Kapodistrian University of Athens and from the 251 Air Force General Hospital as well as from their respective daycare facilities. The overall socioeconomic status of the families was characterised as moderate. The inclusion criteria for this study were children: 1) 2–15 years old, 2) with a non-contributory medical history, and 3) with a basic level of understanding of the Greek language. Children who were not accompanied by their parents on the day of the exam were excluded from the study.

G*power software^13^ was used for power calculation of the caries odds ratio using the app’s % result. To achieve a power of 80% with α = 0.05 and with a medium effect size (0.5), a minimum of 65 children was required per age group.

### Procedure

Written informed consent was received from each participant’s parent after explaining the aim of the study.

#### Questionnaire

Self-reported information about sociodemographics (age, gender, parental education), oral health behaviour (toothbrush frequency, use of fluoride toothpaste and mouthrinse, use of dental floss, and frequency of dental visits), and height/weight was collected via questionnaire. Prior to the study’s initiation, the questionnaire was distributed to 15 parents to assess its comprehension. Each parent filled out a 24-h diet recall with the child’s assistance. Each participant reported the type and quantity of food/beverage consumed, as well as the time and duration of the meal the previous day. A second 24-h recall was collected via the phone from a weekend day.

#### Clinical examination 

Children were clinically examined by a calibrated paediatric dentist using a mirror and a World Health Organzsation (WHO) probe on a dental chair in a dental setting. Caries was assessed only visually according to ICDAS (International Caries Detection and Assessment System) II criteria.^18^ Two thresholds for the calculation of the dmft/DMFT (decayed, missing, filled primary teeth/decayed, missing, filled permanent teeth) indices were used. The 1st threshold (dmft/DMFT) included ICDAS lesions ≥ 3, and the 2nd threshold (d1mft/D1MFT index) was calculated including incipient lesions (ICDAS 1 and 2). Examiner training and calibration to a trained and experienced expert examiner was conducted on 15 healthy patients prior to the initiation of the study. Inter-examiner reliability agreement was κ = 0.86 using Cohen’s Kappa statistics.

#### Diet Application Data

For each child, a score of its dietary cariogenicity was calculated using the app constructed for this study. The application was developed in the fall of 2022 under the name Dental Diet App. Updated versions were made based on the feedback from its use on patients of the Postgraduate Clinic of Paediatric Dentistry, National and Kapodistrian University of Athens. The application was initially made for Androids, but an iOS version was developed later. It is available for use free of charge through the app stores.

#### Collection of data

**Fig 1:**
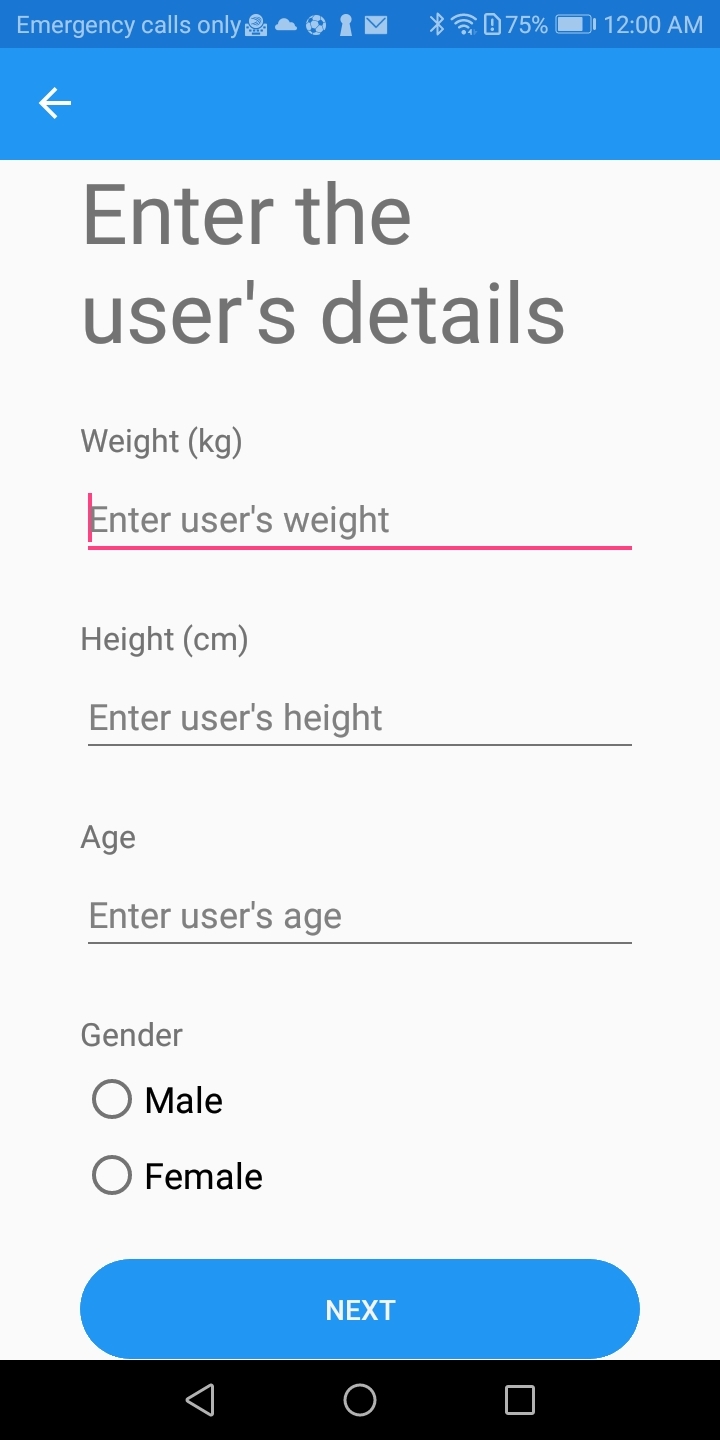
Screen of the app to insert weight, height, age and gender in user’s folder for dietary analysis.

**Fig 2:**
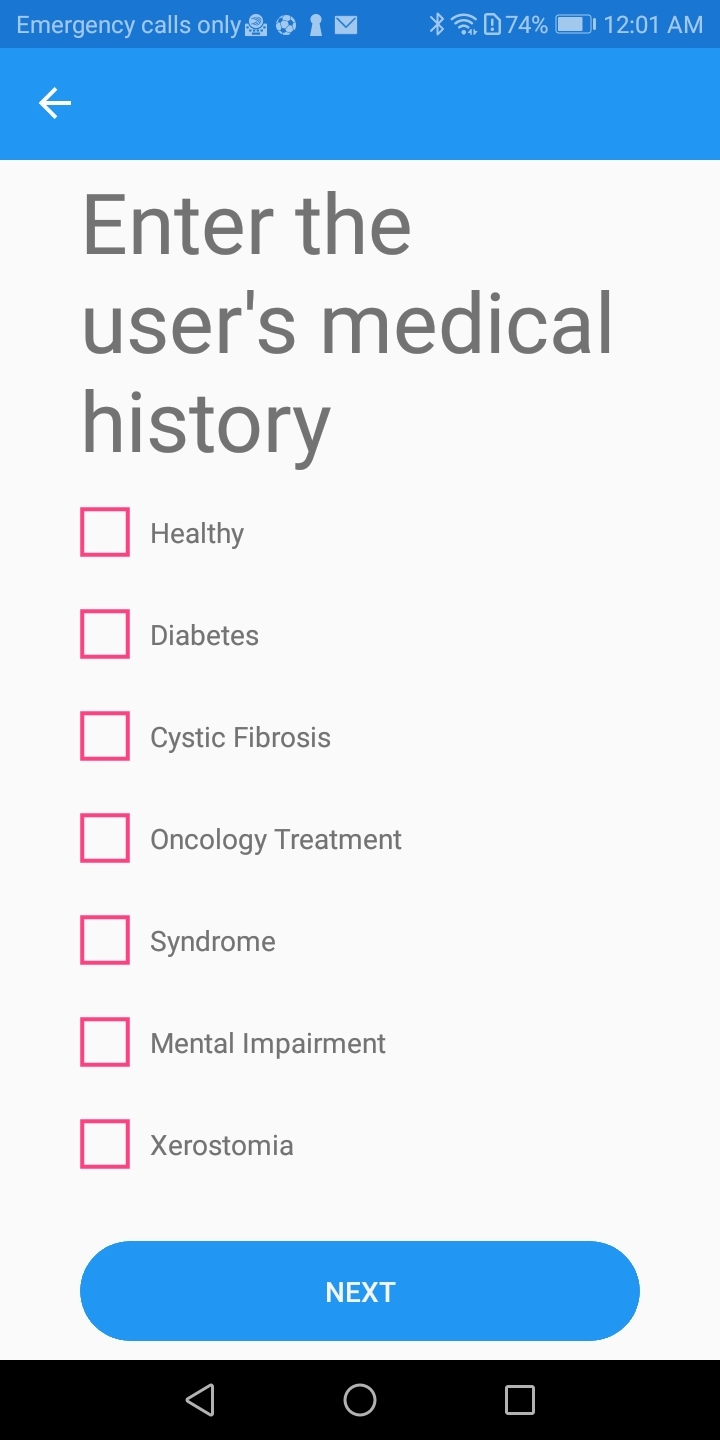
Screen of the app to insert medical history in user’s folder for dietary analysis.

**Fig 3:**
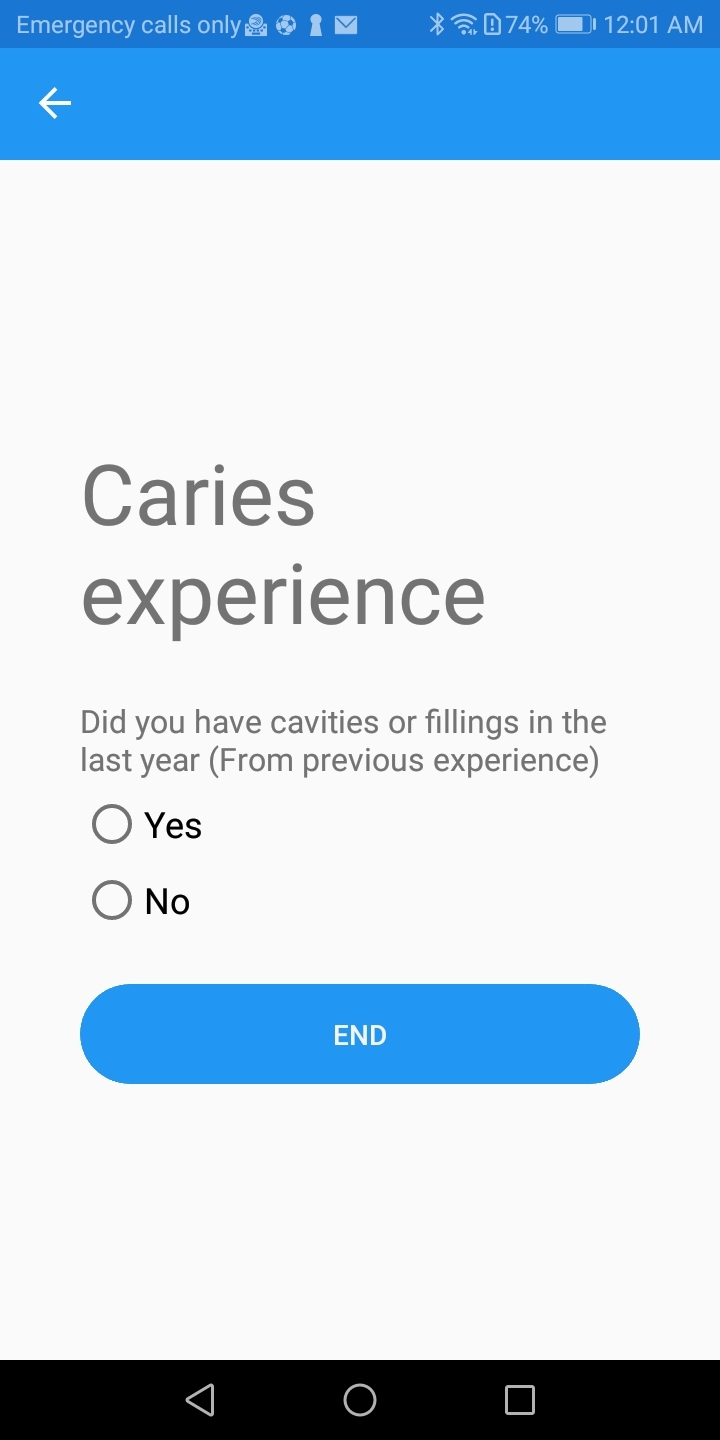
Screen of the app to insert caries experience in user’s folder for dietary analysis.

Initially, patient-related basic information (e.g., age, gender, height, and weight) was entered into the app. Body Mass Index (BMI) was calculated with these data to assess if further dietary advice that could benefit the patient’s general health was required. Specific medical conditions that require a special diet or influence oral health were recorded. Finally, the patient’s clinical oral status, regarding the presence of active caries (ICDAS ≥ 3) or a recent history of caries, was registered (Figs 1–3). This feature can be used as a modifying factor to include the clinician’s judgment in the assessment.

**Fig 4:**
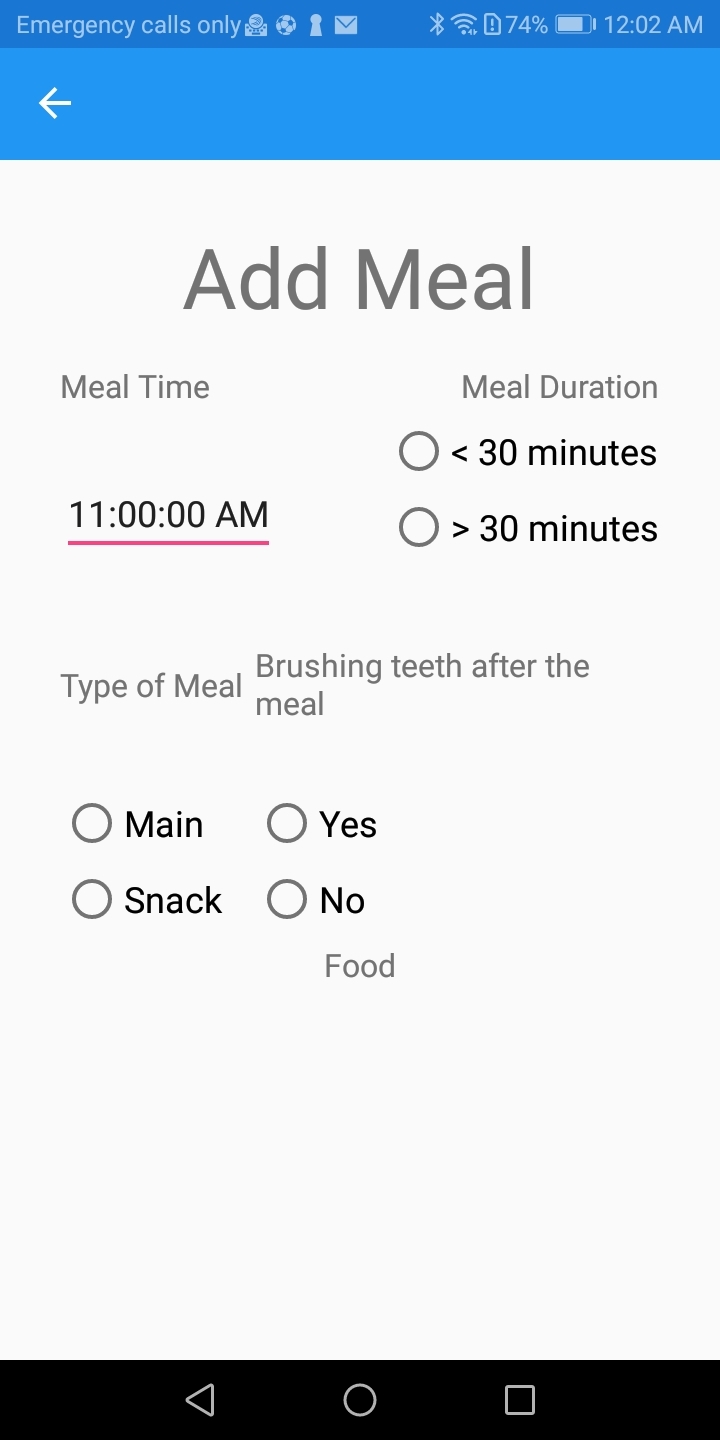
Screen of the app to insert time, duration, and type of each meal and if the user brushed his/her teeth after the meal.

The next step was to insert the 24-h dietary recalls into the app (Fig 4). Participants reported any food items they consumed, the time interval between meals, the duration of the meal, and if they brushed their teeth. Food items were classified in different groups (Table 1). The different food items were selected from the list used in myplate.gov. Additional sugary food items and beverages were added as they are considered important in caries risk. To calculate the cariogenicity, each food item was characterised as low, medium, high, or extremely high based on previously reported data.^10,12,23,27^ Diet may be recorded for several days to increase the accuracy of caries risk estimation. Reporting the diet from one day may not depict the usual dietary habits; thus, multiple days may provide more accurate results.

**Table 1:** Food categories and food items included in each category used in the application (modified from www.myplate.gov)

Beverages	Fruit juice, bottle/carton fruit juice, plain coffee/tea, coffee/tea with sugar, special type coffee, diet soda, soda, energy/sport drink, milkshake, smoothie
Dairy	Cheese, plain milk, milk with honey, milk with sugar, chocolate milk, plain yogurt, frozen yogurt, yogurt with fruit, yogurt with toppings, plain kefir, flavoured kefir, soymilk
Fruit	Apple, apricot, banana, berries, cherries, grapefruit, grape, kiwi, lemon, lime, mango, melon, nectarine, orange, papaya, peach, pear, pineapple, plum, raisins, strawberries, tangerine, watermelon, fruit snack
Grains	Barley, bread, cereal, cereal bar, couscous, crackers, muesli, noodles, oats, pasta, pita, pretzel, quinoa, rice, tortilla
Oils/Sauces	Butter, ketchup, mayo, mustard, oil, peanut butter
Protein foods	Fish/meats/poultry/seafood: catfish, cod, fish, halibut, salmon, sardines, sea bass, snapper, trout, tuna, swordfish, bacon, beef, bison, ground meat, ham, lamb, liver, pork, rabbit, sausage, veal, chicken, duck, eggs, turkey, crab, lobster, mussels, octopus, oysters, scallops, shrimp, squid Nuts/seeds: almonds, cashews, hazelnuts (filberts), mixed nuts, peanut butter, peanuts, pecans, pistachios, walnuts, pumpkin seeds, pumpkin seeds, sesame seeds, sunflower seeds Legumes: all types of beans, incl. tofu
Sweets	Cake, candy, caramels with sugar, sugar-free caramels, chocolate, cookies, doughnut, gum with sugar, sugar-free gum, gummy bears, honey, ice cream, jam, jello, lollipop, marshmallows, pancakes, pastry, popsicle, pudding, sugar, syrup, waffles
Vegetables	Artichokes, asparagus, avocado, broccoli, brussel sprouts, cabbage, carrot, cauliflower, celery, chestnuts, corn, cucmbers, eggplant, ginger, green beans, green peas, kale, lettuce, lima beans, mushrooms, okra, onions, peppers, plantains, potatoes, pumpkin, spinach, squash, sweet potatoes, tomatoes

#### Dietary assessment and analysis

The pH graph and a pie chart were formed by considering the patient’s clinical presence of active caries, oral hygiene, fluoride exposure, and diet.

**Fig 5:**
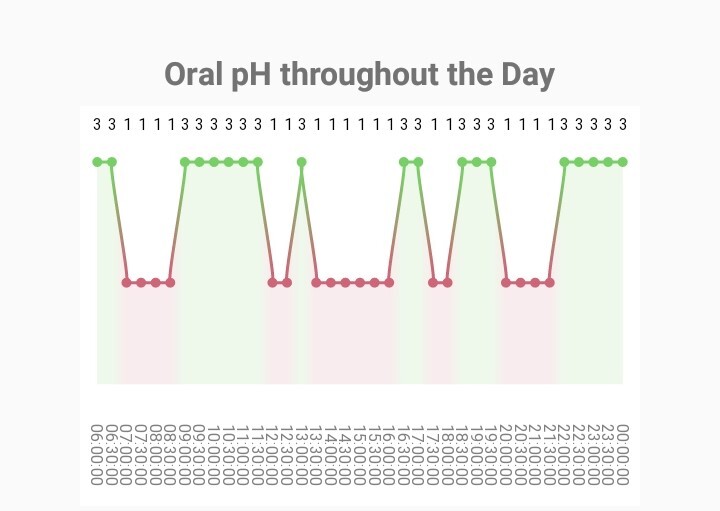
Example of the screen of the app presenting how the oral pH fluctuates after each snack or meal depending on its cariogenicity.

Oral pH is presented with a graph based on the cariogenic risk of the different food items and the behaviour of the patient (Fig 5). Thus, if a food item contained sugar, had a sticky nature, was consumed between meals and the patient did not brush his/her teeth afterwards, the pH would remain at the critical value for a longer time (40 mi). Main meals and non-sugary snacks result in a shorter time (20 min) of lower oral pH. The critical pH is marked by changing from a green to a red area in the diagram, indicating the time that the mouth was exposed to acid and thus demineralisation could occur during that day. Patients with active caries harbour cariogenic bacteria; thus, their diagram has a lower starting point for oral pH.

Protective factors, such as fluoride, help teeth remineralise and can alter the oral pH. For this reason, if the patient marks brushing after meal consumption, oral pH increases immediately thereafter as shown in the pH diagram of the app.

**Fig 6:**
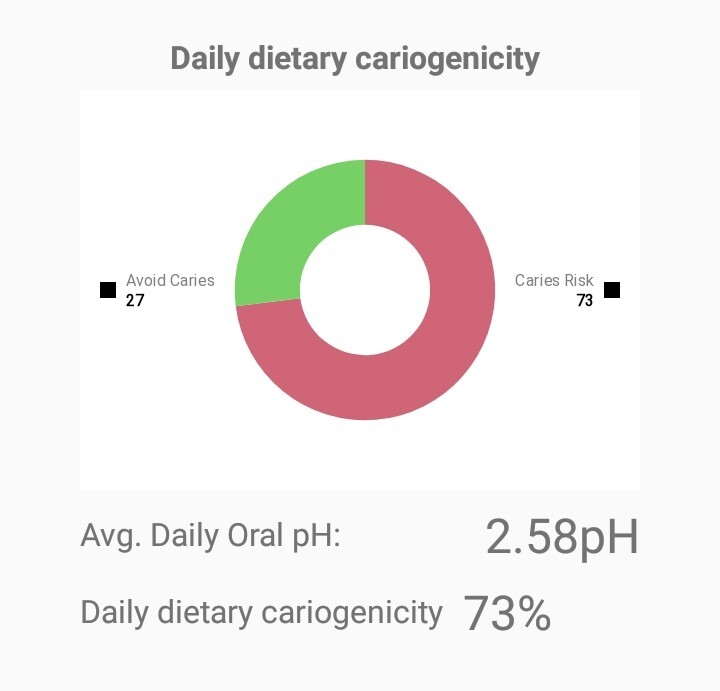
Example of the screen of the app presenting a day’s diet cariogenicity.

In addition, a pie chart indicating the diet’s cariogenicity risk for each day in a percentage is provided (Fig 6). The purpose of the pie chart is to alarm the patient of the cariogenicity of her/his diet, which should motivate changes. Based on caries risk assessment tools, diet is considered to be highly cariogenic when the percentage of the red area in the pie chart is over 61%, moderate with percentages 41–60%, and low with percentages lower than 40%.^8^ In the present study, the pie chart was given to the patient after recording both 24-h recalls, to avoid any bias.

### Statistical Analysis

Data were reported descriptively by calculating frequency, mean, and standard deviation (SD). Student’s t-test and chi-squared test were used to investigate differences in means and proportions between demographic and questionnaire data vs the results of the application. ANOVA and Tukey’s post-hoc test were applied to detect mean differences in cases of variables with more than two groups. Pearson correlations and logistic regression models were used to assess the statistical correlations between caries and the app’s results. Statistically significant differences were investigated at the level of p < 0.05 using Stata (Stata Corp LLC 18.0; College Station, TX).

## Results 

### Descriptive Statistics

The sample’s demographic data are presented in Table 2.

**Table 2:** Sociodemographic data of the sample

Gender	Male	125	50.6
Female	122	49.4
Age in years	2–6	82	33.2
7–11	80	32.4
12–15	85	34.4
Parental educational level	Low	118	47.8
Moderate	55	22.3
High	74	30.0
Total		247	100

Almost half the number of the participants reported brushing their teeth twice daily (48.6%), with the majority using fluoridated toothpaste (83.4%). Of the participants, 64.4% never use dental floss, and 68.1% do not use mouthrinses. Most of the participants reported visiting the dentist once a year (68.4%), 26.3% visit the dentist for emergencies, and 5.3% had never visited the dentist.

Caries prevalence in the total sample had a mean DMFT 1.38 ± 2.10 and dmft 3.22 ± 3.84. The younger children, 2–6 and 7–11 years old, had more decayed than filled teeth (dt = 1.93 ± 3.40 vs ft = 1.24 ± 2.58/ dt + DT = 2.24 ± 2.40 vs ft + FT = 1.8 ± 2.43), while the adolescents had more filled than decayed teeth (DT = 0.71 ± 1.67 vs FT = 1.02 ± 1.70). Detailed data for caries prevalence and severity are presented in Table 3.

**Table 3:** Prevalence of decayed (dt/DT), missing and filled teeth (dmft/DMFT) by age group including incipient carious lesions (d1mft/D1MFT)

Prevalence (primary teeth)
dt	1.93 + 3.40	1.86 + 2.02	0.50 + 0.86
dmft	3.38 + 4.50	3.65 + 3.20	0.71 + 0.85
d1mft	4.21 + 5.09	3.86 + 3.30	1.00 + 1.22
Prevalence (permanent teeth)
DT	0	0.59 + 1.11	0.71 + 1.67
DMFT	0.20 + 0.89	1.29 + 1.79	1.75 + 2.44
D1MFT	0.40 + 0.24	1.88 + 0.21	3.01 + 0.27

### Diet Application Data

**Fig 7:**
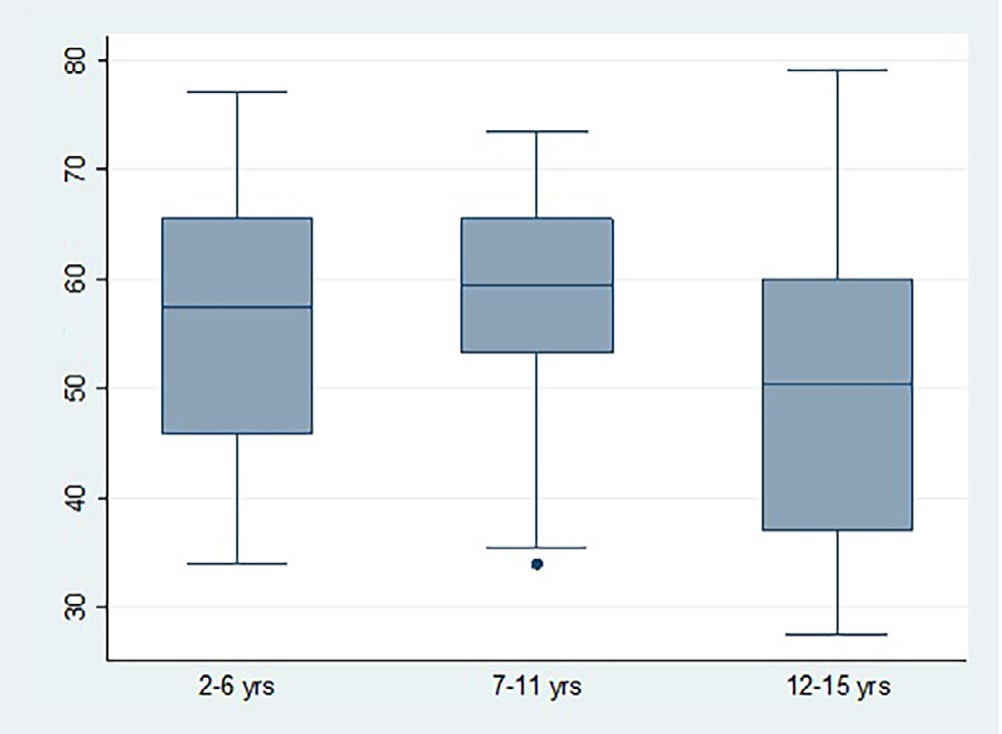
Median and confidence Intervals of percentage values derived from the app by age group.

Overall, 16.2% followed a diet with low cariogenicity, 40.5% followed a diet with moderate cariogenicity, and 43.3% followed a high caries-risk diet based on the app’s assessment. Figure 7 presents the percentage values from the app by age group.

Diet analysis results using the app differed statistically significantly with age (p < 0.001), parental educational level (p < 0.05), and having visited the dentist (p < 0.05). However, the diet did not differ between genders and children with different toothbrushing frequencies. More specifically, Tukey’s test showed that the app’s results differed statistically significantly between young children and the 12-15 years old group, with teenagers having a less cariogenic diet. In regard to parental educational level, children with highly educated parents exhibit a less cariogenic diet according to the app’s results. Finally, patients that never visited the dentist had more highly cariogenic diets.

Correlations between dietary app results and caries are presented in Table 4 separately for each age group. The application’s results were positively associated with the caries level at a statistically highly significant level (p < 0.001) in all age groups.

**Table 4:** Pearson correlations between the results of the app and dental caries by age group

	dmft	dmft	DMFT	DMFT
r	0.4771	0.3761	0.2712	0.3834
p	0.0000*	0.0012*	0.0149*	0.0003*

Based on the logistic regression analyses, children with a high cariogenic diet according to the app had a higher risk of caries (p < 0.001). Moreover, a lower parental education level seemed to be associated with caries risk. Children whose parents both had a lower educational level had much higher odds of caries, compared to children whose parents both had a higher education (p < 0.05). The results were more profound among the younger age groups. A gender difference was found only across the middle age group, where girls were more likely to have caries than were boys (p < 0.05). Younger children brushing their teeth only in the morning were more likely to have caries compared to those who brushed their teeth twice a day, but the results were not statistically significant (p = 0.07).

## Discussion

This newly developed application focuses on the impact of dietary habits on caries and was found to be accurate in the detection of patients with a high cariogenic diet. More specifically, children of all age groups with higher caries prevalence were detected by the app as having a highly cariogenic diet. Thus, this app is a user-friendly tool that can help the dentist and the patient detect a cariogenic diet and eating habits, and modify them to improve their oral health.

The dietary analysis app suggested that teenagers had a higher odds ratio for caries than younger children. This finding can be possibly attributed to the fact that older children usually make their own choices on food/beverage selection,^24^ whereas the diet of children of younger ages is usually controlled by their parents, who also provided the dietary information for the app.

Also, results differed statistically significantly between children with parents of low and high educational levels. This observation has been frequently described in the literature, especially among younger children.^24^ Parents with a higher educational level usually have better knowledge what comprises a healthy diet and access to healthier food choices. Thus, they provide more nutritional and healthier meals to their children.^24^ This finding is even more prominent in young children, as diet is still more controllable by the parents. In terms of gender, primary school girls were more likely to have caries than were boys. This finding was probably random, as there is no evidence suggesting a systematic gender predilection.

The design of the app was based on the existing scientific literature, and multiple factors were taken into consideration for its development. Firstly, specific medical conditions, such as diabetes mellitus, cancer, and cystic fibrosis, require a special diet in relation to meal frequency and type of food.^26^ On these occasions, it is important to follow the suggestions of the treating physician to avoid adverse effects that can threaten the patient’s life. In addition, health conditions that can influence the patient’s oral health, such as asthma, xerostomia, genetic syndromes, etc, are recorded since they indicate a high caries risk for these patients.^36^ For this reason, the medical history of the patient is included in the input of the data provided by the patient, so that the dentist can discuss the individual problems that medically compromised patients may have.

The clinical status of the patient is considered of utmost importance in caries risk estimation, and caries experience remains the best predictor.^3^ Based on this evidence, the patient’s clinical oral status is reported in the app by recording the presence or absence of active caries or the presence of dental restorations in the last year. The clinical judgment of the dental provider is also considered a very good caries risk predictor;^17^ therefore clinicians can use this feature of the app to identify high caries-risk individuals even in cases where active carious lesions are not present.

Diet data is entered in the app using a 24-h dietary recall. An interview with the patient at the 24-h recall is considered a validated method to assess short-term diet.^20^ For more accurate calculations, the app gives the option to input data for more days.^6^ In the present study, two days were recorded for each one of the participants.

Upon input of the dietary data, the app calculates how the oral pH fluctuates in a day. Enamel demineralisation starts when the oral pH drops below 5.5.^22^ After each meal, the pH drops below the critical pH and rises again after 20 min.^28^ The graph of the oral pH is not an actual representation of the patient’s oral acidity levels. Its purpose is to educate the patient on the effects of dietary intake in caries formation and the amount of time during a day that the mouth is exposed to acid.

Patients with active caries harbour cariogenic bacteria, especially of persistent bacteria species.^7^ As a result, their oral pH is lower compared to caries-free patients.^8^ This scientific evidence is depicted in the app, as patients with active caries have a lower oral pH starting point.

To make the graph more accurate and to educate the patient on the benefits of oral hygiene, toothbrushing is taken into consideration when calculating the pH. Fluoride is an element that helps teeth remineralise and results in arresting caries.^14^ It is applied daily on the teeth through toothpaste or a mouthrinse. After each meal, the oral pH drops; however, if fluoride is applied, it can help remineralise the teeth and decrease the caries risk.^14^ For this reason, if the patient marks that he/she brushes or rinses his/her mouth with fluoride products, oral pH increases right after meal consumption.

The clinical presence of active caries, oral hygiene status, fluoride exposure and diet, which are considered the most important factors in caries risk assessment,^3^ are taken into consideration to construct a caries-risk pie chart for the patient. As mentioned before, this software is not intended to be used as a caries risk tool, but as a dietary educational tool to alarm the patients on the cariogenicity of their diet and thus motivate them to modify the dietary behaviour.

Dietary advice in the dental office usually includes general instructions to avoid sweets and sugary snacks, but this method has limited effectiveness.^27^ However, individualised counseling and tailored suggestions can lead to better results in dietary modification.^25^ Specifically in children, preschool children usually experience caries as a result of nocturnal lactation or bottle feeding combined with the early introduction of sugary snacks, while the types of food consumed are more controlled by the parents.^34^ Schoolchildren tend to be more independent in their food choices, and often skip breakfast, resulting in more frequent snacking through the day.^11^ In teenagers, on the other hand, caries development is usually related to sugary beverage consumption.^4^ This application offers the clinician the ability to provide specific suggestions that can be individualised based on the diet already followed by the patient, helps to propose healthier dietary choices and adjust the number of meals/snacks based on the chart’s findings.

Technology-based diet tools have several advantages, such as real-time reporting, which decreases recall bias.^20^ Also, they provide a list of food items that help individuals report all of the food items consumed. In addition, they are more attractive especially to the younger age groups, and can motivate the individual to change behaviours by sending notifications. They are easy to use and are available to everyone.^20^

The limitations of the current application are that the patient may find it difficult to interpret the results without the help of a dental professional. Moreover, updated versions of the app may be required based on the cultural differences of diet between countries. A limitation of this study was that it included only healthy children. Further studies will be needed to test the app’s accuracy in adult populations. Moreover, the sample consisted of patients from dental clinics or associated daycare facilities and is not representative of the general population, although representativeness is not a requirement for the investigations of associations between exposures and outcomes. Finally, this study followed a cross-sectional design. In the future, a prospective study could be designed to test the effects of dietary advice in this population.

## Conclusions

This app was found to provide accurate results for the estimation of diet’s cariogenicity in this sample of children. Multiple benefits could be found in using this app for dietary analysis in the dental office. It offers the possibility to increase patient awareness and knowledge of diet by using graphs, while also enabling specific individualised advice to the patient on necessary dietary alterations. Also, it saves clinical time, as the information can be entered by an office member prior to the patient’s appointment, and dietary advice can be given by any member of the dental team after minimal training. In addition, the results can be printed out and given to the patient for reference. In the modern era of technology, this app could capture the interest and enhance the motivation of young patients to perform the dietary assessment and comply with the preventive program and advice.
